# The contribution of activated astrocytes to Aβ production: Implications for Alzheimer's disease pathogenesis

**DOI:** 10.1186/1742-2094-8-150

**Published:** 2011-11-02

**Authors:** Jie Zhao, Tracy O'Connor, Robert Vassar

**Affiliations:** 1 Department of Cell & Molecular Biology, Northwestern University Feinberg School of Medicine, Chicago, Illinois, 60611, USA

**Keywords:** Aβ, APP, Astrocyte, BACE1, β-secretase, Cytokine, IFN-γ, Neuroinflammation, oligomer, TNF-α

## Abstract

**Background:**

β-Amyloid (Aβ) plays a central role in Alzheimer's disease (AD) pathogenesis. Neurons are major sources of Aβ in the brain. However, astrocytes outnumber neurons by at least five-fold. Thus, even a small level of astrocytic Aβ production could make a significant contribution to Aβ burden in AD. Moreover, activated astrocytes may increase Aβ generation. β-Site APP cleaving enzyme 1 (BACE1) cleavage of amyloid precursor protein (APP) initiates Aβ production. Here, we explored whether pro-inflammatory cytokines or Aβ42 would increase astrocytic levels of BACE1, APP, and β-secretase processing, implying a feed-forward mechanism of astrocytic Aβ production.

**Methods:**

Mouse primary astrocytes were treated with combinations of LPS, TNF-α, IFN-γ, and IL-1β and analyzed by immunoblot and ELISA for endogenous BACE1, APP, and secreted Aβ40 levels. Inhibition of JAK and iNOS signaling in TNF-α+IFN-γ-stimulated astrocytes was also analyzed. In addition, C57BL/6J or Tg2576 mouse astrocytes were treated with oligomeric or fibrillar Aβ42 and analyzed by immunoblot for levels of BACE1, APP, and APPsβsw. Astrocytic BACE1 and APP mRNA levels were measured by TaqMan RT-PCR.

**Results:**

TNF-α+IFN-γ stimulation significantly increased levels of astrocytic BACE1, APP, and secreted Aβ40. BACE1 and APP elevations were post-transcriptional at early time-points, but became transcriptional with longer TNF-α+IFN-γ treatment. Despite a ~4-fold increase in astrocytic BACE1 protein level following TNF-α+IFN-γ stimulation, BACE1 mRNA level was significantly decreased suggesting a post-transcriptional mechanism. Inhibition of iNOS and JAK did not reduce TNF-α+IFN-γ-stimulated elevation of astrocytic BACE1, APP, and Aβ40, except that JAK inhibition blocked the APP increase. Finally, oligomeric and fibrillar Aβ42 dramatically increased levels of astrocytic BACE1, APP, and APPsβsw through transcriptional mechanisms, at least in part.

**Conclusions:**

Cytokines including TNF-α+IFN-γ increase levels of endogenous BACE1, APP, and Aβ and stimulate amyloidogenic APP processing in astrocytes. Oligomeric and fibrillar Aβ42 also increase levels of astrocytic BACE1, APP, and β-secretase processing. Together, our results suggest a cytokine- and Aβ42-driven feed-forward mechanism that promotes astrocytic Aβ production. Given that astrocytes greatly outnumber neurons, activated astrocytes may represent significant sources of Aβ during neuroinflammation in AD.

## Background

The neuropathology of Alzheimer's disease (AD) is characterized by the development of extracellular deposits of senile amyloid plaques that are mainly composed of the β-amyloid peptide (Aβ). AD pathogenesis is likely to involve elevated cerebral Aβ levels that in turn cause neuroinflammation and neurodegeneration, ultimately leading to dementia through a cascade of neurotoxic events [1-5]. Marked by focal activation of microglia and astrocytes in the vicinity of amyloid plaques, AD-associated inflammation has been widely described by pathological examination of brain tissue from AD patients and transgenic mouse models [3,6-16]. It has therefore received much attention in the analysis of AD pathological progression [17-19]. The resulting neuroinflammatory processes usually involve the release from activated glia of a number of potentially neurotoxic molecules, including reactive oxygen species, nitric oxide, and pro-inflammatory chemokines and cytokines such as interleukin-1β (IL-1β), tumor necrosis factor-α (TNF-α), and interferon-γ (IFN-γ). Excessive levels of these mediators are apt to induce neuronal damage through a variety of mechanisms in AD and other neurodegenerative disorders [20]. Although the inflammatory processes in AD have been well studied, the amyloidogenic potential of glial cells under pro-inflammatory conditions and the mechanisms involved have been relatively unexplored.

Neurons are believed to be the major source of Aβ in normal and AD brains [21,22]. Aβ is a proteolytic product of amyloid precursor protein (APP) resulting from sequential cleavages by the β- and γ-secretase enzymes [2]. The transmembrane aspartic protease BACE1 (β-site APP-cleaving enzyme 1; also known as Asp2 and memapsin 2) has been identified as the β-secretase and is therefore the key enzyme that initiates Aβ peptide generation [23-27]. Among specific cell populations in the CNS, neurons express higher levels of BACE1 than glial cells like astrocytes, indicating that astrocytes are less likely to be significant generators of Aβ under normal conditions [23,28]. However, it should be noted that AD may take decades to develop and progress, and astrocytes outnumber neurons by over five-fold in the brain [29,30]. Together, these data suggest the possibility that the generation of astrocyte-derived Aβ, even if low on a per-cell basis, could contribute significantly to cerebral Aβ levels and exacerbate amyloid pathology over time in AD.

A limited number of studies to date have investigated the effects of pro-inflammatory cytokine and Aβ stimulation on BACE1 and APP levels and β-secretase processing of APP in astrocytes. APP levels have been reported to be elevated by certain pro-inflammatory conditions in mouse brain and in human neuroblastoma and non-neuronal cells, as well as in human astrocyte cultures, suggesting the potential for amyloidogenic APP processing associated with pro-inflammatory conditions [31-34]. The synergistic effects of TNF-α and IFN-γ on promoting Aβ production have been demonstrated for cultured cells including astrocytes [33,35,36]. In addition, it has been reported that IFN-γ alone stimulated BACE1 expression and β-secretase cleavage in human astrocytoma cells and astrocytes derived from Tg2576 transgenic mice that overexpress human APP with the Swedish familial AD mutation (APPsw), but its effect on Aβ production was not investigated [37,38]. A subsequent study suggested that the IFN-γ-stimulation activated BACE1 gene transcription via the JAK/STAT signaling pathway in astrocytes [39]. Other studies in APP transgenic mice have provided further support for the involvement of TNF-α and IFN-γ in the development of AD-related amyloid pathology and memory dysfunction [40,41]. One report showed that TNF-α and IFN-γ stimulation increased Aβ production in Tg2576 transgenic astrocytes [40]. However, no study to date has explored the effects of TNF-α and IFN-γ on endogenous wild-type APP, BACE1 and Aβ in astrocytes, which may be more relevant to AD than transgenically overexpressed mutant APP.

Conversely, other studies have shown that Aβ *itself *is able to stimulate astrocytes to secrete pro-inflammatory molecules *in vitro *and *in vivo *[42-45]. Oligomers of Aβ42, the 42 amino acid fibrillogenic form of Aβ, disrupt synaptic function and activate astrocytes [1,2,42,43,46]. Fibrillar Aβ42, which is a primary component of amyloid plaques, also causes astrocyte activation [43]. Together with the cytokine cycle of neuroinflammation, these results suggest that a feed-forward loop may operate during AD whereby cytokines stimulate the production and secretion of Aβ in astrocytes, and then astrocytic Aβ in turn promotes further cytokine release and astrocytic Aβ generation [4,17]. This is a compelling hypothesis, but direct evidence in support of it has been limited thus far.

Here, to investigate whether activated astrocytes could be significant sources of Aβ during AD neuroinflammation and whether an amyloidogenic astrocytic feed-forward mechanism may exist, we treated cultured primary wild-type C57BL/6J or Tg2576 mouse astrocytes with pro-inflammatory cytokine combinations or Aβ42 oligomers and fibrils and measured levels of BACE1, APP, secreted Aβ40, or APPsβsw, the β-secretase cleavage product. We observed that cytokines, especially combinations containing TNF-α+IFN-γ, raised the levels of endogenous BACE1 and APP in C57BL/6J astrocytes and promoted the secretion of astrocytic Aβ40. Inhibitor treatments suggested that iNOS signaling was not involved in cytokine-stimulated astrocytic BACE1, APP, and Aβ40 elevations, although JAK signaling appeared to have a role in the endogenous astrocytic APP increase. Similar to the effects of cytokine stimulation, Aβ42 oligomers and fibrils elevated levels of endogenous BACE1 and APP in C57BL/6J astrocytes, and increased β-secretase cleavage of APPsw in Tg2576 astrocytes. The astrocytic APP and BACE1 elevations for cytokine or Aβ42 stimulations appeared in some cases to involve combined transcriptional and post-transcriptional mechanisms, depending on the stimulation. Overall, our results support the hypothesis that cytokine- and Aβ42-stimulated astrocytes could contribute significantly to the total burden of cerebral Aβ in AD, potentially through elevated astrocytic β-secretase processing of APP under neuroinflammatory conditions. Moreover, the similar effects of cytokine or Aβ42 stimulation on astrocytic β-secretase processing suggest a feed-forward mechanism that might promote Aβ generation in astrocytes.

## Methods

### Materials and reagents

The bacterial endotoxin LPS purchased from Sigma-Aldrich (St. Louis, MO) was from *Salmonella typhimurium*. Stock solutions were prepared with sterile Dulbecco's phosphate-buffered saline (D-PBS) (Invitrogen-Gibco; Carlsbad, CA) at a concentration of 1 mg/ml. The recombinant murine cytokines TNF-α, IL-1β, and IFN-γ were purchased from R&D Systems (Minneapolis, MN) and reconstituted in sterile 0.1% bovine serum albumin (BSA; Sigma) in D-PBS at a concentration of 10, 5, 50 μg/ml, respectively. iNOS inhibitor (1400W; Catalog # ALX-270-073) was procured from Alexis Biochemicals (San Diego, CA); JAK inhibitor (Catalog # 420099) was obtained from EMD-Calbiochem (San Diego, CA). Aβ42 peptide was purchased from American Peptide (Sunnyvale, CA). Antibodies used for immunoblotting and fluorescence immunocytochemistry are listed in Table 1. The RNeasy Mini Kit from Qiagen (Valencia, CA) was applied for astrocyte RNA isolation and real-time PCR experiments.

**Table 1:** Primary antibodies used for immunoblotting and immunocytochemistry procedures

	Host	Clone	Dilution	Source
***Monoclonal***				
Anti-APP A4	mouse	22C11	1:5000	Chemicon
Anti-β-actin	mouse	AC-15	1:20,000	Sigma
Anti-F4/80	rat	CI:A3-1	1:1000 (fluor. ICC^a^)	Serotec
Anti-GFAP	mouse	G-A-5	1:3 × 10^9^ 1:10^8 ^(fluor. ICC^a^)	Sigma
Anti-IL-1β	mouse	3ZD	1:50,000	National Cancer Institute
***Polyclonal***				
Anti-BACE1 (PA1-757)	Rabbit	------	1:1000	Affinity BioReagents
Anti-NOS2 (M-19)	Rabbit	------	1:1000	Santa Cruz Biotechnology

^a^Abbreviation: fluor. ICC = fluorescence immunocytochemistry

### Primary astrocyte culture

The wild-type C57BL/6J and Tg2576 transgenic mice used in this study were purchased from Taconic (Germantown, NY) and colonies of these mice were kept in the Northwestern University Center for Comparative Medicine animal facilities. All animal procedures were in strict accordance with the NIH *Guide for the Care and Use of Laboratory Animals *and were approved by the Northwestern University Animal Care and Use Committee.

Mouse primary astrocyte cultures were established from cerebral cortices of newborn mouse pups as previously described with some modifications [47]. In brief, postnatal day 1-3 (P1-3) wild-type C57BL/6J mouse brain cortices were harvested in ice-cold D-PBS, and meninges and blood vessels were removed. Tissues were digested in 0.25% Trypsin containing 0.1% EDTA (Mediatech; Herndon, VA) at 37°C for 15 min, cells were dispersed by gentle trituration, and seeded in Dulbecco's modified Eagle's medium (DMEM; Mediatech) with 10% fetal bovine serum (FBS; Hyclone; Logan, UT) and 1% antibiotic solution (100 U/ml penicillin-100 μg/ml streptomycin; Invitrogen-Gibco) in 75 cm^2 ^T-flasks at a density of 1 cortex/flask. Cells were grown in the 37°C incubator with 5% CO_2_. After 12 days *in vitro*, the mixed glial cultures became a confluent monolayer, and cells were then detached by trypsinization and re-plated at 1 × 10^6 ^cells/well in 6-well plates for pro-inflammatory agent treatments. For Aβ42 treatments, astrocytes were re-plated at 5 × 10^5 ^cells/well in 12-well plates. The purity of astrocytes (> 90%) in the mixed glial cultures with this method was verified using fluorescence immunocytochemistry by staining with anti-glial fibrillary acidic protein (GFAP; astrocyte marker) and anti-F4/80 (microglia marker) antibodies (Table 1; data not shown).

### LPS and pro-inflammatory cytokine treatments

LPS was selected as a control in this study due to its well-established features as a potent pro-inflammatory agent. Twenty-four hours after re-plating, mouse primary astrocytes were treated with fresh growth media containing pro-inflammatory agents, either individually or in specific combinations at concentrations described previously [36,38]. Single-agent treatments were: LPS (1 μg/ml), TNF-α (30 ng/ml), IL-1β (10 ng/ml), IFN-γ (20 ng/ml); combination treatments were: LPS+IFN-γ, TNF-α+IFN-γ, TNF-α+IL-1β+IFN-γ (concentrations same as for single treatments). After 24, 48, or 96 h of treatment, media were collected, cells were washed two times in ice-cold D-PBS, and then cells were lysed in buffer containing 40 mM Tris-HCl, pH 6.8, 2% sodium dodecyl sulfate (SDS), 10% glycerol, 0.02% sodium azide with freshly added protease inhibitor cocktail (EMD-Calbiochem) for 10 min on ice followed by brief sonication. Both media and cell lysate samples were stored at -80°C until analysis.

### Inhibitor treatments

Inhibitors were prepared as concentrated stock solutions according to respective manufacture's instructions. The final concentrations of inhibitors in media applied to astrocytes were the following: 1400W (iNOS inhibitor): 1, 8, and 50 μM; JAK inhibitor: 1, 5, and 20 μM. Inhibitors were added to culture medium 30 min prior to stimulation of cells with TNF-α+IFN-γ for 96 h. Conditioned medium and cell protein extraction following the treatments were harvested as above.

### Aβ42 preparation and treatment

Human Aβ42 (American Peptide; Sunnyvale, CA) oligomers and fibrils were prepared as previously described with minor modifications [48]. Briefly, Aβ42 was dissolved in hexafluoroisopropanol (HFIP), lyophilized, dissolved in dimethylsulfoxide (DMSO; Sigma) to a concentration of 5 mM, and then diluted to make 100 μM stocks either with ice-cold phenol red-free Ham's F12 medium (Biosource; Rockville, MD) for making oligomers, or with 10 mM HCl at room temperature for making fibrils. Before treating cells, the 100 μM Aβ42 stocks, and vehicle controls lacking Aβ42, were incubated for 24 h on ice for oligomers or at 37°C for fibrils. One day prior to Aβ42 treatments, primary astrocytes were washed twice with D-PBS, and changed into serum-free media. Specifically, G-5 supplement (Invitrogen-GIBCO) was used at 1% to replace FBS in the growth media for primary astrocytes. Twenty-four hours after serum-free media was applied, 100 μM Aβ42 oligomer and fibril stocks were added to astrocyte cultures at a final concentration of 10 μM in the media, and cells were treated for 6, 24, 48, or 96 h.

### Immunofluorescence microscopy

Mouse primary astrocytes were plated onto coverslips at 5 × 10^5 ^cells/well in 12-well plates and were then treated with 10 μM oligomeric Aβ42 for 24 h, as described above. Coverslips were then washed two times in D-PBS, fixed in 4% paraformaldehyde/D-PBS, and blocked and permeabilized in 1% heat inactivated normal goat serum/D-PBS/0.1% Triton-X100. Astrocytes were stained with anti-APP antibody 22C11 (Chemicon) at 1:200 dilution, washed, and incubated with goat anti-mouse Alexa 594 antibody (Invitrogen) at 1:500 dilution. Following a final wash and mount with anti-fade, astrocytes were imaged with a fluorescence Nikon Eclipse E800 microscope and Spot advanced digital camera (Diagnostic Instruments, Sterling Heights, MI).

### Immunoblot analysis

Protein concentrations of the cell lysates were measured using the BCA protein assay kit from Pierce (Rockford, IL). Equal amounts (10-20 μg) of protein were separated on 4-12% NuPAGE Bis-Tris gels in MOPS buffer (Invitrogen) and transferred to Millipore Immobilon-P polyvinylidene difluoride (PVDF) membranes (Fisher Scientific). The blots were cut into strips (based on the size of the protein of interest), blocked in 5% nonfat dry milk made in Tris-buffered saline with 0.1% Tween 20 (TBST; Sigma; modified form), pH 8.0, for 1 h at room temperature (RT) or overnight at 4°C, and then incubated with primary antibodies recognizing APP (overnight at 4°C), BACE1 (2 h at RT), GFAP (overnight at 4°C), or IL-1β(overnight at 4°C). After washing in TBST, blots were incubated in horseradish peroxidase (HRP)-conjugated goat anti-mouse (for APP, 1:5000; for GFAP, 1:30,000; for IL-1β, 1:5000; 1 h at RT) or goat anti-rabbit (for BACE1, 1:5000; 1 h at RT) secondary antibodies. Finally, blots were developed using enhanced chemiluminescence (ECL) Plus detection reagents (Amersham Biosceinces; Piscataway, NJ), and digitally imaged using a Kodak Image Station 440C. Some blots were processed in stripping buffer containing 62.5 mM Tris-HCl, pH 6.7, 2% SDS and 115 mM β-mercaptoethanol at 55°C for 30 min, and then re-probed with anti-NOS2 (iNOS) and anti-β-actin antibodies followed by incubation in HRP-conjugated goat anti-rabbit (1:10,000) and goat anti-mouse (1:20,000) secondary antibodies, respectively, as described above. For relative quantification of immunosignals, band intensities recorded with the Kodak Image Station were expressed as percent of vehicle control within each individual experiment.

### RNA isolation and real-time PCR

Astrocytes of C57BL/6J brains were treated with TNF-α or IFN-γ, either singly or in combination for 6, 24, or 96 h, and their RNA was isolated using the RNeasy Mini kit (Qiagen) and real-time PCR procedures were carried out as described before with some modifications [49]. Briefly, cells were homogenized in guanidine isothiocyanate (GITC)-containing buffer (RLT buffer) supplied in the RNeasy Mini kit with addition of 1% β-mercaptoethanol. Following determination of RNA concentration, 1 μg of total RNA from each sample was used for first-strand cDNA synthesis using the Invitrogen SuperScript III reverse transcription system. cDNA was amplified using quantitative real-time PCR with Assays-on-Demand premixed Taqman primer/probe set for mouse APP and BACE1 mRNAs (Applied Biosystems; Foster City, CA) and analyzed using an Applied Biosystems 7900HT sequence analyzer with the relative quantification method normalized against 18S rRNA (Applied Biosystems). All samples were run in triplicate and averages were determined, and then were expressed as percent of vehicle control within each individual experiment before means and SEMs were acquired.

### Mouse Aβ40 ELISA

Endogenous mouse Aβ40 secreted into the culture media by C57BL/6J primary astrocytes following pro-inflammatory stimulation was measured by sandwich enzyme-linked immunosorbant assay (ELISA), using reagents from Biosource International (Camarillo, CA). In brief, 96-well NUNC MaxiSorp immunoplates (VWR) were coated with mouse monoclonal anti-mouse Aβ capture antibody (clone 252Q6; Catalog # AMB0062) diluted at 1:100 in 0.1 M sodium carbonate coating buffer overnight at 4°C. Plates were then blocked in 200 μl/well of 2% BSA made in D-PBS for 1 h at RT followed by incubation with native rodent Aβ1-40 peptide (Catalog # 03-189) standards [dissolved in Dimethyl Sulfoxide (DMSO) (Sigma) at 1000 μg/ml as a stock, then diluted to final concentrations of 0, 7.8, 15.6, 31.3, 62.5, 125, 250, and 500 pg/ml in growth media] or cell culture media samples, together with detection antibody rabbit anti-Aβ40 (Catalog # 44-348) diluted in blocking buffer at 1 μg/ml for 2 h at RT with rocking. After extensive washing, HRP-conjugated goat anti-rabbit secondary antibody (Catalog # ALI4404) (1:2000 in blocking buffer) was added to the plates for 1 h at RT, followed by chromogen for 15-30 min. The reaction was terminated by addition of stop solution immediately before the absorbance was read at 450 nm on a microplate spectrophotometer (Spectra Max 250; Molecular Devices). Unless otherwise indicated, all reagents above were added at 100 μl/well in each step, and were obtained from a human Aβ40 ELISA kit (Biosource International, Catalog # KHB3481). Aβ40 levels in the media were normalized to total protein in the respective cell lysates and expressed as pg/mg total protein or percent of vehicle control within each individual experiment.

### Statistical analysis

Relative quantification of APP and BACE1 immunoblot bands was performed using Kodak 1D 3.6 image analysis software. At least three independent experiments using C57BL/6J or Tg2576 primary astrocyte cultures pooled from ~1-3 cortices for each experiment were analyzed. Statistical significance was determined using two-tailed *t*-test (two samples assuming equal variances) with Microsoft Excel. The data are presented as the mean ± standard error of the mean (SEM), and p < 0.05 was considered significant.

## Results

### Pro-inflammatory cytokine combinations increase astrocytic BACE1, APP, and Aβ

To investigate whether activated astrocytes increase amyloidogenic APP processing under pro-inflammatory conditions, we treated primary astrocytes cultured from neonatal C57BL/6J mouse pups with pro-inflammatory agents LPS, TNF-α, IL-1β, and IFN-γ, both individually and in the combinations LPS+IFN-γ, TNF-α+IFN-γ, TNF-α+IL-1β+IFN-γ. Numerous studies have reported that these pro-inflammatory cytokines are elevated in AD brain [reviewed in [3,4,17,20]]. In addition, we used LPS as a control, since it has been well studied as a stimulus that strongly activates astrocytes both *in vitro *and *in vivo*. After astrocyte cultures were treated for 24, 48, and 96 h, cell lysates were prepared for immunoblot analysis of BACE1, APP, and activation markers iNOS and pro-IL-1β, and conditioned media was harvested for mouse Aβ40 measurement.

**Figure 1:**
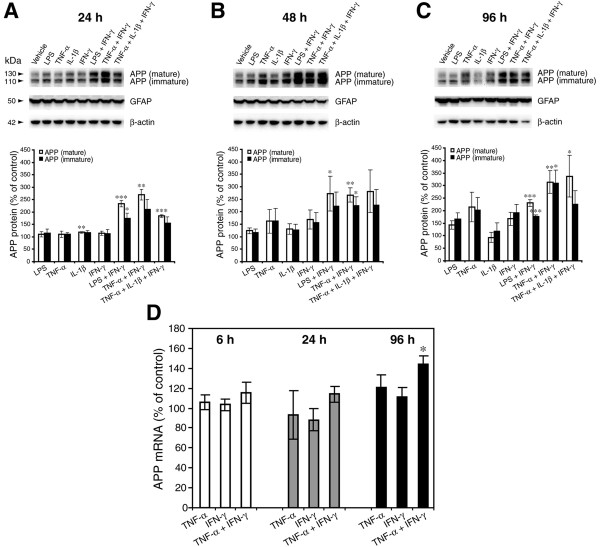
**Combinations of pro-inflammatory cytokines elevate endogenous APP levels in mouse primary astrocyte cultures**. (A-C) Cultured wild-type C57BL/6J mouse primary astrocytes were stimulated with the indicated pro-inflammatory agents (alone and combinations) for 24 (A), 48 (B), or 96 h (C). Cell lysates were then prepared and analyzed for APP, GFAP, and β-actin by immunoblot. Upper panels show APP immunoblot images and lower histograms represent quantifications of APP immunoblot signals expressed as percent of vehicle control. The mature APP band at 130 kDa and the immature APP band at 110 kDa are indicated by the arrowheads. GFAP and β-actin immunosignals served as loading controls. Note that cytokine combinations including TNF-α and IFN-γ were generally more potent at increasing endogenous APP levels in astrocytes over time in culture, raising APP levels to ~300% of control. (D) C57BL/6J mouse primary astrocyte cultures were stimulated with TNF-α, IFN-γ, TNF-α+IFN-γ, or vehicle control for the indicated times and analyzed for endogenous APP mRNA levels by TaqMan quantitative RT-PCR. Histograms represent quantifications of APP mRNA levels expressed as percent of vehicle control. Note that only TNF-α+IFN-γ-stimulated astrocytes at 96 h exhibited a statistically significant increase in APP mRNA. Statistical analysis for A-D was performed by two-tailed *t*-test based on a normal distribution of the data. Significance indicates comparison to individual vehicle control within each measurement and each time point (n = 3; *p < 0.05, **p < 0.01, ***p < 0.001). Error bars, standard error of the mean (SEM).

The anti-APP antibody 22C11 labeled both mature (130 kDa) and immature (110 kDa) glycosylated forms of full-length APP (Figure 1A-C), and showed that endogenous APP levels in astrocytes appeared increasingly higher in a time-dependent manner following stimulation with all tested individual pro-inflammatory agents when compared to controls, with the exception of IL-1β. The pro-inflammatory cytokine combinations TNF-α+IFN-γ and TNF-α+IFN-γ+ IL-1β produced robust elevations of astrocytic APP levels, reaching ~150-350% of vehicle controls for all time points. In vehicle-treated cells, basal levels of the ~130 kD mature APP were consistently lower than those of the ~110 kD immature form at all time points. Interestingly, although the cytokine combinations increased both mature and immature APP forms, the magnitudes of the elevations tended to be larger for mature than immature APP (Figure 1A). Together these results suggested that cytokine combination stimulation may enlarge the pool of mature APP substrate for subsequent amyloidogenic processing by BACE1 in astrocytes.

To determine whether the cytokine-stimulated elevation in astrocytic APP protein level could have been the result of increased APP gene transcription, we prepared stimulated primary astrocyte cultures as described above and measured APP mRNA levels by real-time TaqMan quantitative RT-PCR (Figure 1D). Cytokine stimulation did not significantly alter astrocytic APP mRNA levels relative to those of vehicle controls, with the exception that APP mRNA levels in astrocytes treated for 96 h with TNF-α+IFN-γ were elevated to ~150% of control values. These data suggested that a significant proportion of the early cytokine-stimulated increases in APP level could be the result of a post-transcriptional mechanism. However, increased APP gene transcription or longer APP mRNA half-life might also contribute to the cytokine-induced APP elevation, especially for longer stimulation times with cytokine combinations.

**Figure 2:**
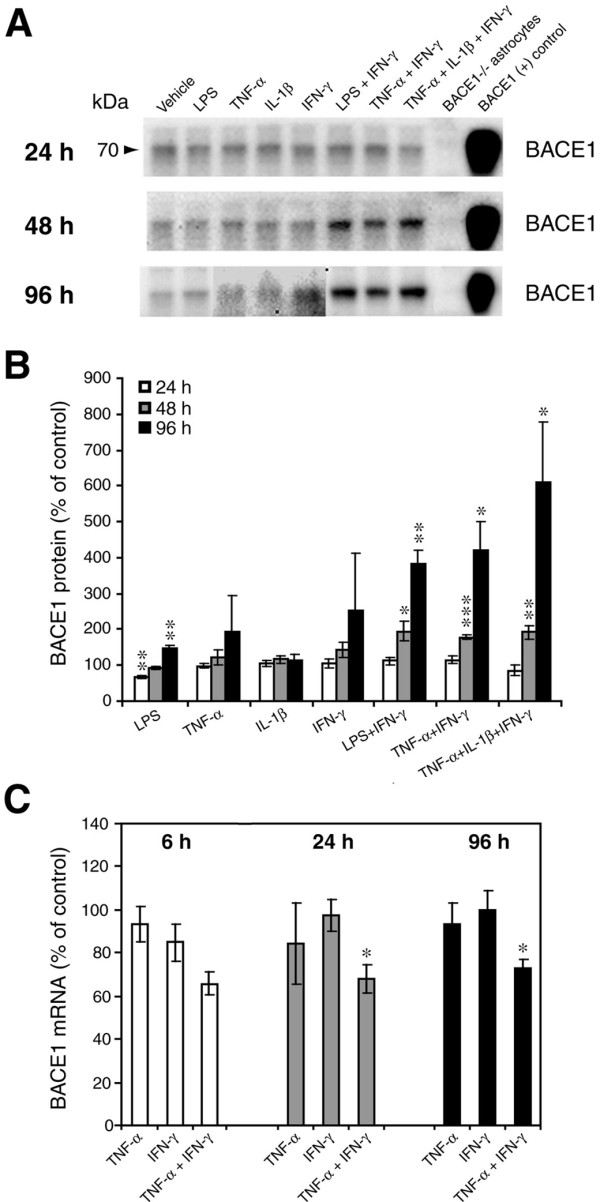
**Combinations of pro-inflammatory cytokines elevate endogenous BACE1 levels in mouse primary astrocyte cultures**. (A, B) Cell lysates of cytokine-stimulated mouse primary astrocytes analyzed in Fig. 1A-C were analyzed for BACE1 by immunoblot. (A) BACE1 immunoblot images. The mature BACE1 band at 70 kDa is indicated by the arrowhead. Pro-inflammatory agents (alone and combinations) and stimulation times are indicated. Cell lysate from un-stimulated BACE1^-/- ^primary astrocytes was used as a negative control, while cell lysate from a stable BACE1-overexpressing HEK-293 cell line was used as a positive control. GFAP and β-actin immunosignals were loading controls as in Fig 1A-C. (B) Histograms represent quantifications of BACE1 immunoblot signals in (A) expressed as percent of vehicle control. Note that cytokine combinations including TNF-α and IFN-γ were generally more potent at increasing endogenous BACE1 levels in astrocytes over time in culture, raising BACE1 levels to ~600% of control. (C) mRNAs prepared from cytokine-stimulated primary astrocytes in Fig 1D were analyzed for endogenous BACE1 mRNA levels by TaqMan quantitative RT-PCR. Histograms represent quantifications of BACE1 mRNA levels expressed as percent of vehicle control. Note that astrocytic BACE1 mRNA levels were significantly reduced by TNF-α+IFN-γ stimulation (C), even though BACE1 protein levels were increased several fold in similarly treated astrocytes (B). Statistical analysis was the same as described in Fig. 1. Significance indicates comparison to individual vehicle control within each measurement and each time point (n = 2-3; *p < 0.05, **p < 0.01, ***p < 0.001). Error bars, SEM.

Since BACE1 cleavage of APP initiates Aβ generation, we also measured endogenous BACE1 levels in the same primary astrocytes that were stimulated by the pro-inflammatory agents above. By using lysates of primary astrocytes from BACE1^-/- ^mice as negative controls in immunoblots (Figure 2A, lanes 9), we clearly demonstrated that un-stimulated astrocytes express low but readily detectable levels of mature BACE1 (~70 kDa) [50]. Following 24 h of stimulation, none of the treatments resulted in notable changes in BACE1 level with the exception of LPS alone, which unexpectedly reduced BACE1 levels by a slight amount (Figure 2B), although this effect was transient. Treatments with individual cytokines did not significantly alter BACE1 levels at any time point. Importantly, however, cytokine combinations caused moderate (~200%) and strong (~400-600%) BACE1 elevations at 48 h and 96 h, respectively, as compared to vehicle. This dramatic rise in BACE1 level with cytokine combinations suggested that pro-inflammatory conditions in AD could elevate astrocytic BACE1 and potentially increase amyloidogenic APP processing in astrocytes.

We then investigated whether the cytokine-stimulated increase in astrocytic BACE1 protein level was potentially the result of enhanced BACE1 gene expression. Primary astrocyte cultures treated as above were prepared for TaqMan quantitative RT-PCR to measure BACE1 mRNA levels (Figure 2C). Stimulation with the individual cytokines TNF-α or IFN-γ did not produce significant alterations of astrocytic BACE1 mRNA levels. In contrast, the cytokine combination TNF-α+IFN-γ unexpectedly caused a ~20-30% reduction in BACE1 mRNA level in astrocytes (Figure 2C). Thus, despite a large (~4-fold) increase in BACE1 protein level by 96 h of TNF-α+IFN-γ stimulation, BACE1 mRNA levels were significantly decreased, strongly suggesting that a post-transcriptional mechanism was responsible for the cytokine-stimulated rise in astrocytic BACE1.

Thus far, our results indicated that cytokine combinations could markedly increase levels of endogenous APP and BACE1 in astrocytes. We next sought to determine whether the cytokine-stimulated APP and BACE1 increases would correlate with greater astrocytic Aβ production. Toward this end, we collected conditioned media (CM) from the cytokine-stimulated astrocytes described above and measured endogenous secreted mouse Aβ40 in CM by sandwich ELISA. It is of note that pathogenic Aβ42 is generated in proportion to Aβ40, yet Aβ40 levels are higher for robust quantification. Thus, changes in Aβ40 level faithfully reflect alterations of Aβ42 level.

**Figure 3:**
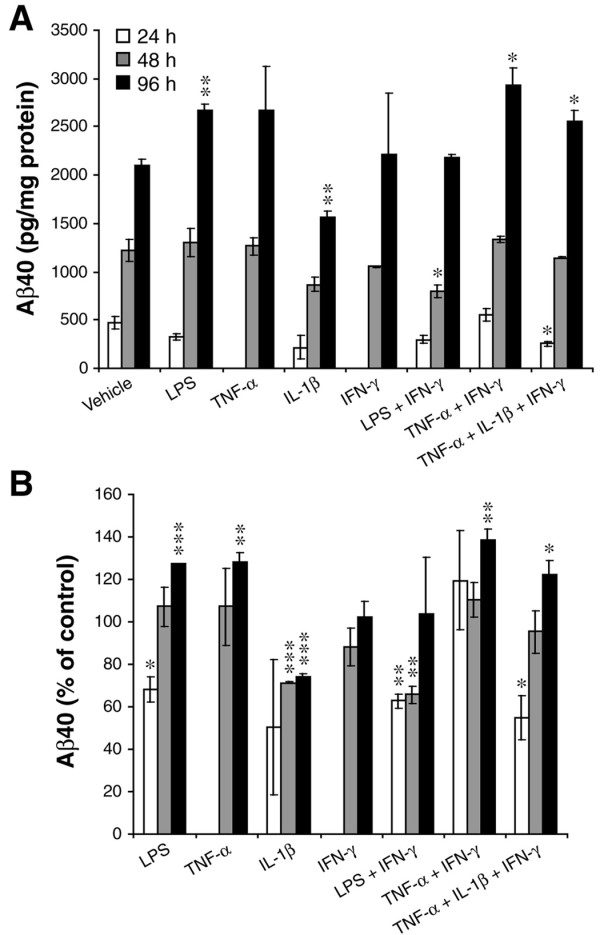
**Combinations of pro-inflammatory cytokines elevate endogenous secreted Aβ40 levels in conditioned media from mouse primary astrocyte cultures**. Conditioned media (CM) of cytokine-stimulated mouse primary astrocytes analyzed in Fig. 1A-C and Fig. 2A, B were harvested after 24, 48, or 96 h of proinflammatory agent (individual and combinations) stimulation and analyzed for endogenous mouse Aβ40 levels by sandwich ELISA. The amount of Aβ40 in CM was expressed as pg/mg total protein in the cell lysate (A) or as percent of vehicle control (B). Note that TNF-α+IFN-γ stimulation was overall more potent at increasing astrocytic secreted Aβ40 levels, while IL-1β reduced Aβ40 levels at all time points. Statistical analysis was the same as described in Fig. 1. Significance indicates comparison to individual vehicle control within each time point (n = 3; *p < 0.05, **p < 0.01). Error bars, SEM.

As expected, endogenous astrocytic Aβ40 levels increased in CM from 24 h to 96 h irrespective of treatment (Figure 3A). However, the accumulation rates and the absolute values of secreted Aβ40 varied depending on the treatment. Stimulations with LPS, TNF-α, TNF-α+IFN-γ, and TNF-α+IL-1β+IFN-γ all caused secreted Aβ40 levels to increase to ~120-140% of vehicle control, but only after 96 h of treatment (Figure 3B). IL-1β alone, on the other hand, resulted in decreased levels of secreted Aβ40 at all time points. Aβ40 levels were also reduced by LPS at 24 h, LPS+IFN-γ at 24 h and 48 h, and TNF-α+IL-1β+IFN-γ at 24 h. Thus, treatments that included IL-1β, either added exogenously or induced endogenously (i.e., by LPS treatment), caused a decrease in Aβ40 level in CM from astrocytes at early (LPS, LPS+IFN-γ, TNF-α+IL-1β+IFN-γ) or all (IL-1β) time points. Nevertheless, prolonged stimulation for 96 h with pro-inflammatory cytokine combinations resulted in elevated levels of endogenous secreted astrocytic Aβ40.

**Figure 4:**
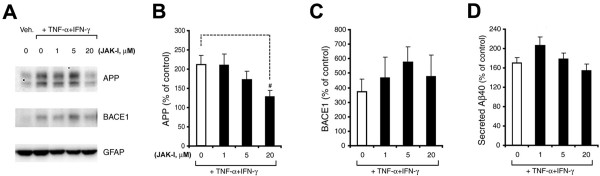
**Inhibition of JAK blocks the TNF-α+IFN-γ-stimulated increase in endogenous APP level in mouse primary astrocytes, but not that of BACE1 or secreted Aβ40**. Cultured wild-type C57BL/6J mouse primary astrocytes were pre-treated for 30 min with JAK Inhibitor I at 0, 1, 5, and 20 μM and then stimulated for 96 h with TNF-α+IFN-γ. Cell lysates and CMs were harvested and analyzed for endogenous levels of APP (A, B) and BACE1 (A, C) by immunoblot and secreted Aβ40 by ELISA (D). (A) Immunoblot images for APP, BACE1, and GFAP (loading control) signals. Lanes with lysates of astrocytes that received inhibitor treatments and TNF-α+IFN-γ stimulation are indicated. (B-D) Histograms represent quantifications of signals for APP (B) and BACE1 (C), as well as that of secreted Aβ40 (D) expressed as percent of un-stimulated vehicle control. TNF-α+IFN-γ treatment alone significantly elevated astrocytic APP, BACE1, and secreted Aβ40 levels over un-stimulated vehicle controls ("0" bars in B-D). In contrast, JAK Inhibitor I significantly reduced the TNF-α+IFN-γ-stimulated increase in astrocytic APP level ("20" bar in B; #: *p *< 0.05, n = 3). Statistical analysis was the same as described in Fig. 1. Error bars, SEM.

Next, we sought to gain initial insights into potential signaling pathways that might raise levels of endogenous APP, BACE1, and Aβ in astrocytes. Stimulation with TNF-α+IFN-γ was used because this combination robustly elevated astrocytic APP, BACE1, and secreted Aβ. We first investigated the JAK pathway (Figure 4), which has been implicated in IFN-γ receptor signaling. Mouse primary astrocytes cultures were pre-treated for 30 min. with 0, 1, 5, or 20 μM JAK Inhibitor (JAK-I) followed by exposure to TNF-α+IFN-γ in the continued presence of inhibitor. After 96 h of stimulation, cell lysates and CMs were harvested for APP and BACE1 immunoblot (Figure 4A-C) and Aβ40 ELISA analyses (Figure 4D), respectively. JAK-I reduced the TNF-α+IFN-γ-stimulated increase in astrocytic APP level in a dose-dependent manner (Figure 4A, B), but it did not block the elevations in astrocytic BACE1 (Figure 4A, C) or secreted Aβ40 (Figure 4D). Unexpectedly, JAK-I treatment with 1 μM and 5 μM appeared to elevate secreted Aβ40 and BACE1 levels above 0 μM JAK-I, respectively, but these increases were not significant. Although it is unclear why JAK-I elevated astrocytic Aβ40 and BACE1 at certain concentrations but not others, it is important to emphasize that JAK inhibition did not prevent the TNF-α+IFN-γ-stimulated increase in BACE1 level, suggesting that JAK signaling may play a synergistic but not essential role in the TNF-α+IFN-γ-stimulated BACE1 elevation. Given that JAK-I reduced the TNF-α+IFN-γ-stimulated increase in astrocytic APP, it is not completely clear why secreted Aβ40 levels were also not reduced by JAK inhibition. Secreted Aβ40 levels appeared slow to change in response to TNF-α+IFN-γ stimulation (Figure 3), so we speculate that secreted Aβ40 could have become significantly reduced with JAK-I treatment times longer than 96 h. This is supported by an observed downward trend in secreted Aβ40 with higher JAK-I concentrations (Figure 4D). Regardless, our JAK-I results overall indicate that JAK signaling, at least in part, may play a role in elevating astrocytic APP levels and this might contribute to secreted Aβ, although JAK signaling does not appear to contribute to an essential degree to BACE1 levels in astrocytes.

**Figure 5:**
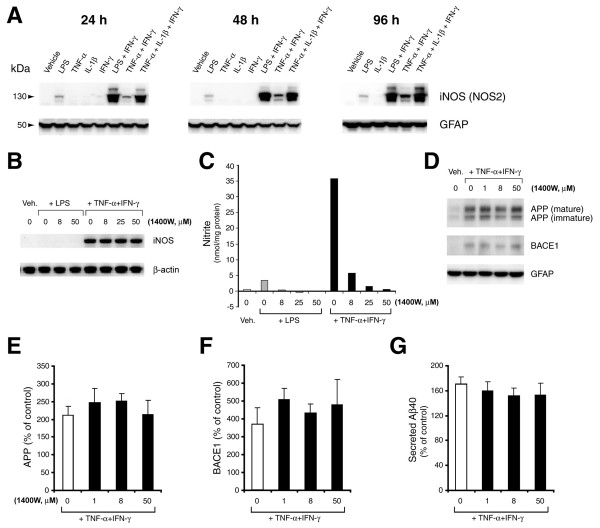
**Inhibition of iNOS does not block the TNF-α+IFN-γ-stimulated increases in levels of endogenous APP, BACE1, or secreted Aβ40 in mouse primary astrocyte cultures**. (A) Cell lysates of cytokine-stimulated mouse primary astrocytes analyzed in Fig. 1A-C were prepared for iNOS (NOS2) immunoblot. The 130 kDa iNOS band is indicated by the arrowhead. Pro-inflammatory agents (alone and combinations) and stimulation times are shown. GFAP immunosignal served as a loading control. Note that iNOS levels were more strongly induced in astrocytes that were stimulated by pro-inflammatory agent combinations but not by single agent treatments, with the exception of LPS. (B-G) Cultured wild-type C57BL/6J mouse primary astrocytes were pre-treated for 30 min with the iNOS inhibitor 1400 W at 0, 8, 25, or 50 μM and were then stimulated for 96 h with TNF-α+IFN-γ. Cell lysates and CMs were harvested and analyzed for endogenous levels of iNOS (B), nitrite production in CM (C), APP (D, E) and BACE1 (D, F) by immunoblot and secreted Aβ40 by ELISA (G). β-actin or GFAP immunosignals served as loading controls. Histograms in E-G represent quantifications of signals for APP (E) and BACE1 (F), as well as that of secreted Aβ40 (G) expressed as percent of un-stimulated vehicle control. TNF-α+IFN-γ treatment alone significantly elevated astrocytic APP, BACE1, and secreted Aβ40 levels over un-stimulated vehicle controls ("0" bars in E-G). Note that iNOS inhibition did not significantly block the TNF-α+IFN-γ-stimulated increases in levels of astrocytic APP, BACE1, or secreted Aβ40. Error bars, SEM (n = 3).

We also investigated signaling through iNOS (NOS2), an inflammatory mediator induced by cytokine stimulation, to explore its potential involvement in amyloidogenic APP processing in astrocytes (Figure 5). Cell lysates from stimulated astrocytes were analyzed by immunoblot to determine iNOS levels. Paralleling the previously observed increases in endogenous APP, BACE1, and Aβ40 levels, iNOS levels were dramatically induced by pro-inflammatory agent combinations at all time points in stimulated astrocytes (Figure 5A). With the exception of the bacterial endotoxin LPS, no single-agent treatment induced appreciable iNOS expression in these cells. These results demonstrated that the elevations of endogenous APP, BACE1, and Aβ40 correlated well with the induction of iNOS in cytokine-stimulated astrocytes.

To determine whether iNOS played a role in the elevation of astrocytic APP, BACE1, and Aβ40 levels, we pre-treated primary astrocytes cultures with the iNOS inhibitor 1400 W for 30 min followed by stimulation with TNF-α+IFN-γ for 96 h (Figure 5B-G). As expected, 1400 W pre-treatment strongly inhibited iNOS activity as demonstrated by dose-dependent suppression of astrocytic nitrite production (Figure 5C) without affecting iNOS protein levels (Figure 5B). Immunoblot analysis of cell lysates revealed that the TNF-α+IFN-γ-stimulated rise in astrocytic APP and BACE1 was not significantly blocked by iNOS inhibition (Figure 5D-F). However, ELISAs of CMs showed that iNOS inhibition slightly blunted the increase in secreted Aβ40 levels to ~90% of control values (Figure 5G), but this effect was not statistically significant. These results suggested that iNOS signaling might make a small contribution to cytokine-stimulated increases in astrocytic secreted Aβ, but it may do so via a mechanism that is independent of effects on APP and BACE1 expression.

### Aβ42 increases astrocytic BACE1, APP, and β-secretase processing

It has been posited that AD may involve a "vicious cycle" that becomes self-perpetuating once it is started [3,51]. However, direct evidence for this hypothesis has been difficult to obtain. Given that we observed that Aβ secretion was increased in cytokine-stimulated astrocytes, and that astrocytic cytokine release was induced by Aβ, we investigated the possibility of an astrocytic vicious cycle involving an Aβ-stimulated feed-forward loop [42,44]. Specifically, we sought to determine whether oligomers and fibrils of Aβ42, the putative pathogenic agent in AD, could elevate endogenous APP, BACE1, and β-secretase cleavage of APP in astrocytes. If so, astrocytes might represent a significant source of Aβ production in AD, and understanding the associated mechanism(s) could potentially identify novel astrocyte-specific Aβ-lowering therapeutic strategies.

**Figure 6:**
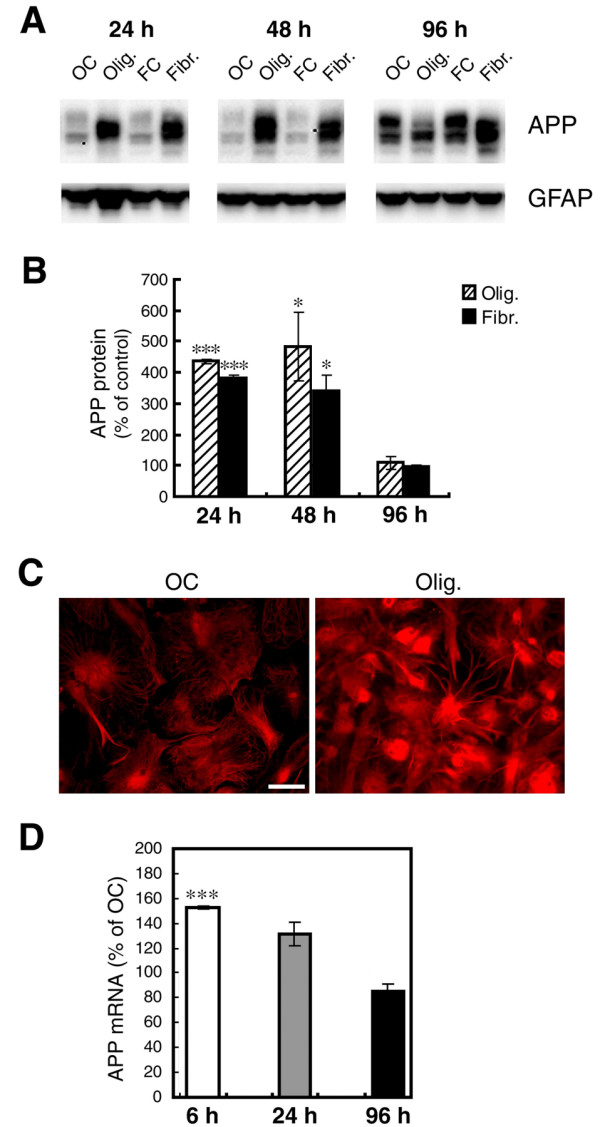
**Oligomeric and fibrillar Aβ42 increase levels of endogenous APP in mouse primary astrocyte cultures**. C57BL/6J mouse primary astrocyte cultures were treated with 10 μM oligomeric or fibrillar Aβ42. Following treatment, cells were harvested for either APP immunoblot (A, B), immunofluorescence microscopy (C), or mRNA quantification by TaqMan RT-PCR (D). Treatment times are indicated, or are 24 h (C). Olig., Aβ42 oligomer treatment; Fibr., Aβ42 fibril treatment; OC, oligomer vehicle control; FC, fibril vehicle control. GFAP immunosignals served as loading controls. Aβ42 oligomers and fibrils dramatically increased APP protein levels to ~400-500% of controls at early treatment times (A-C) that correlated with an early rise in APP mRNA level (D). Levels of both APP protein and mRNA were transiently elevated by Aβ42, and returned to normal by 96 h. Asterisks indicate significant differences as compared to vehicle controls (*: *p *< 0.05; ***: *p *< 0.001; n = 3). Error bars, SEM.

To gain insight into these questions, we cultured primary astrocytes from the brains of neonatal C57BL/6J or Tg2576 mouse pups and then treated astrocyte cultures with either oligomeric or fibrillar Aβ42 prepared as previously described [48]. Following treatment, cell lysates were harvested and analyzed for levels of endogenous APP and BACE1 protein and mRNA, and APPsβ, the BACE1-cleaved APP ectodomain fragment. For C57BL/6J wild-type primary astrocytes, APP immunoblots revealed that both Aβ42 oligomers and fibrils stimulated a dramatic 400-500% rise in endogenous APP protein level after 24 h of Aβ42 treatment, as compared to oligomeric or fibrillar vehicle controls (Figure 6A, B). This Aβ42-stimulated APP increase remained elevated at 48 h of Aβ42 treatment, but APP levels returned to vehicle control levels by 96 h of treatment (Figure 6A, B). Immunofluorescence microscopy with anti-APP antibody 22C11 confirmed this robust increase in astrocytic APP level following 24 h of oligomeric Aβ42 treatment (Figure 6C). These results suggested that Aβ42, irrespective of its aggregation state, was capable of strongly inducing the expression of endogenous astrocytic APP, at least up to 48 h of exposure under the culture conditions that we tested.

To determine whether the Aβ42-stimulated astrocytic APP elevation was potentially the result of a transcriptional mechanism, we grew C57BL/6J primary astrocyte cultures, treated them with Aβ42 and then isolated mRNA and measured APP mRNA levels with TaqMan quantitative RT-PCR (Figure 6D). Since both oligomeric and fibrillar Aβ42 caused similar increases of APP level in astrocytes, we focused on Aβ42 oligomer-treated astrocytes because the mechanisms of APP elevation for both forms of Aβ42 seemed likely to be the same. In addition, mounting evidence suggests that oligomeric forms of Aβ may be more toxic than the fibrillar Aβ found in amyloid plaques, and therefore the former is of considerable therapeutic interest. We observed a rapid, highly significant ~160% increase in APP mRNA level following only 6 h of oligomeric Aβ42 treatment, compared to vehicle control (Figure 6D). By 24 h of treatment, APP mRNA levels were returning to normal, and by 96 h oligomer- and vehicle-treated astrocytic APP mRNA levels were the same (Figure 6D). These results demonstrated that the Aβ42-stimulated astrocytic APP elevation was the result of either elevated APP gene transcription or increased APP mRNA stability.

**Figure 7:**
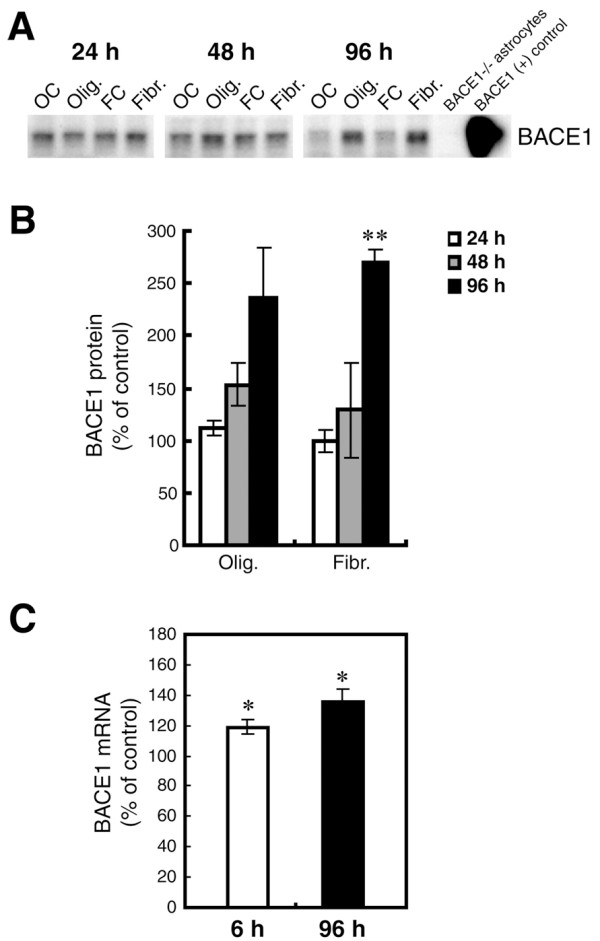
**Oligomeric and fibrillar Aβ42 increase levels of endogenous BACE1 in mouse primary astrocyte cultures**. Lysates of C57BL/6J mouse primary astrocyte cultures treated with 10 μM oligomeric or fibrillar Aβ42 from Fig. 6 were prepared for BACE1 immunoblot (A, B) or mRNA quantification by TaqMan RT-PCR (C). Treatment times are indicated. Olig., Aβ42 oligomer treatment; Fibr., Aβ42 fibril treatment; OC, oligomer vehicle control; FC, fibril vehicle control. Cell lysate from un-treated BACE1^-/- ^primary astrocytes was used as a negative control, while cell lysate from a stable BACE1-overexpressing HEK-293 cell line was used as a positive control. GFAP immunosignals were loading controls as in Fig. 6A. Aβ42 oligomers and fibrils elevated BACE1 protein levels at 48 h and 96 h of treatment (A, B). BACE1 levels peaked at nearly 300% of control at 96 h that correlated with a rise in BACE1 mRNA level (C). Unlike APP, levels of both BACE1 protein and mRNA did not return to normal but remained elevated at 96 h. Asterisks indicate significant differences as compared to vehicle controls (*: *p *< 0.05; **: *p *< 0.01; n = 3). Error bars, SEM.

Next, we sought to determine whether Aβ42 treatment could increase endogenous astrocytic BACE1 protein levels. Cell lysates isolated from the oligomeric and fibrillar Aβ42-treated C57BL/6J primary astrocytes used for APP immunoblots (Figure 6A) were analyzed by immunoblot for BACE1 levels (Figure 7A). In contrast to the APP immunoblot results, neither oligomeric nor fibrillar Aβ42 treatment caused a significant increase in BACE1 level after 24 or 48 hours of stimulation, although a slight upward trend was observed at 48 h compared to controls (Figure 7B). However, a strong ~300% increase in BACE1 level was apparent after 96 h of treatment with Aβ42 oligomers and fibrils. While the fibrillar Aβ42-induced astrocytic BACE1 elevation was robust (p < 0.01), the oligomer-induced BACE1 increase did not reach statistical significance because of high immunoblot signal variability (Figure 7B). However, BACE1 mRNA levels were significantly elevated by oligomer treatment (Figure 7C), suggesting that the BACE1 protein increase was likely real. These results suggested that Aβ42 could increase levels of endogenous BACE1 in astrocytes regardless of Aβ42 aggregation state.

To determine whether the Aβ42-stimulated increase of astrocytic BACE1 was possibly the result of a transcriptional mechanism, we performed BACE1 TaqMan RT-PCR on mRNA isolated from the oligomeric Aβ42-treated primary astrocytes used for the APP mRNA measurements described above. Aβ42 oligomers caused a significant increase in the level of astrocytic BACE1 mRNA as early as 6 h of treatment, an effect that persisted for at least 96 h (Figure 7C). Although relatively small (~140% of control), this early and long-lasting increase in BACE1 mRNA level was likely responsible for the elevation of BACE1 protein that we observed by immunoblot (Figure 7A, B). A substantial lag period existed between the increases of BACE1 mRNA and protein levels, most likely because the small BACE1 mRNA elevation resulted in a slow accumulation of BACE1 protein in astrocytes.

Thus far, our experiments demonstrated that Aβ42 oligomers and fibrils could raise both endogenous APP and BACE1 levels in astrocytes. However, they did not address whether this elevation of substrate and enzyme could lead to greater Aβ production. Unfortunately, we were unable to directly measure endogenous astrocytic Aβ production in Aβ42-treated astrocytes because the Aβ42 treatment interfered with ELISA measurements of astrocytic Aβ that was secreted into conditioned media (CM) (not shown). To overcome this problem, we designed an experiment to directly measure BACE1 processing of APP, which positively correlates with Aβ production in cells. In this experiment, we investigated the effects of Aβ42 oligomers and fibrils on primary astrocytes cultured from Tg2576 transgenic mice that overexpress APPsw, which is a superior BACE1 substrate as compared to wild-type APP [52]. As a consequence, Tg2576 neurons and astrocytes exhibit rates of APPsw amyloidogenic processing and Aβ production that are substantially higher than those of non-transgenic cells.

**Figure 8:**
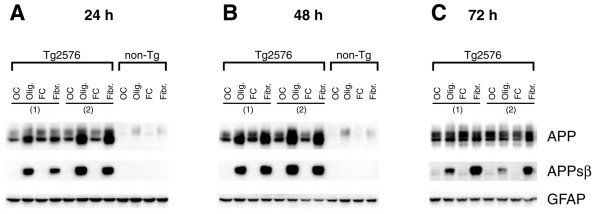
**Oligomeric and fibrillar Aβ42 increase levels of APPsβsw in Tg2576 transgenic mouse primary astrocytes**. Tg2576 or non-transgenic (non-Tg) littermate mouse primary astrocyte cultures were treated with 10 μM oligomeric or fibrillar Aβ42. Following treatment, cells were harvested for APP and APPsβsw immunoblots. Treatment times are indicated. Olig., Aβ42 oligomer treatment; Fibr., Aβ42 fibril treatment; OC, oligomer vehicle control; FC, fibril vehicle control. The numbers in parentheses indicate primary astrocytes cultured from different Tg2576 mouse pups. Immunosignals identified by the anti-APP antibody 22C11 clearly demonstrated that Tg2576 astrocytes overexpress transgene-derived APPsw (A-C, upper row). Note that Tg2576 APPsw migrates faster than non-Tg astrocytic APP on SDS-PAGE because the Tg2576 transgene expresses the 695 amino acid form of APP, while endogenous APP expressed by astrocytes is the 751 amino acid form. Aβ42 oligomers and fibrils caused both transgenic APPsw and endogenous astrocytic APP to become elevated at 24 and 48 h (A, B). The APPsw increase largely returned to control levels by 72 h for Tg2576 astrocytes (C). Most importantly, oligomeric and fibrillar Aβ42 treatment dramatically induced β-secretase processing of APPsw to generate robust levels of APPsβsw in Tg2576 astrocytes (A-C, center row), as indicated by immunosignals produced by an antibody that recognizes the BACE1-cleaved C-terminal neo-epitope of APPsβ containing the Swedish mutation. The APPsβsw elevation remained high for the duration of the experiment. GFAP immunosignals served as loading controls (A-C, lower row).

BACE1 cleavage of APPsw generates an N-terminal ectodomain fragment of APPsw that is named APPsβsw. To measure levels of APPsβsw, we generated an antibody that specifically recognizes the cleaved C-terminal neo-epitope of APPsβsw following BACE1 processing [23]. We used this anti-APPsβsw neo-epitope antibody to perform immunoblots of cell lysates from Tg2576 primary astrocytes that were stimulated with Aβ42 oligomers or fibrils for 24, 48, or 72 h (Figure 8). Tg2576 astrocytes expressed several fold more APP than non-transgenic astrocytes, demonstrating that the Tg2576 transgene promoter was active in astrocytes. Moreover, stimulation with Aβ42 oligomers and fibrils caused levels of both transgenic and endogenous APP to significantly increase in Tg2576 and non-transgenic astrocytes, respectively, at 24 and 48 h time-points (Figure 8), similar to results obtained with Aβ42-treated C57BL/6J astrocytes (Figure 6). Most importantly, robust APPsβsw signals on immunoblots indicated that Aβ42 stimulation of Tg2576 astrocytes caused dramatic increases in BACE1 cleavage of APPsw at all treatment time-points. Both oligomeric and fibrillar Aβ42 stimulation elevated APPsβsw levels to similar extents at the earlier time-points (Figure 8A, B), although the potency of Aβ42 oligomers appeared to decrease somewhat relative to Aβ42 fibrils by 72 h of treatment (Figure 8C). APPsβsw signals were absent in immunoblot lanes of lysates from vehicle control-treated Tg2576 astrocytes (Figure 8A-C), indicating that Aβ42 may have induced non-amyloidogenic astrocytes to initiate BACE1 cleavage of APP. Taken together, these results demonstrated that Aβ42 oligomers and fibrils are not only capable of elevating levels of astrocytic APP and BACE1, but they could also increase BACE1 cleavage of APP in astrocytes, a prerequisite of Aβ synthesis.

## Discussion

Are astrocytes a significant source of Aβ in AD? Is a feed-forward "vicious cycle" involved in AD pathogenesis? These are underappreciated yet critical questions that have important mechanistic and therapeutic implications for AD. Several studies have attempted to address certain aspects of these problems, but our study is the first to integrate these questions and address whether specific cytokine combinations and forms of Aβ42 are capable of increasing amyloidogenic APP processing and Aβ generation in astrocytes. We first determined that pro-inflammatory cytokine combinations including TNF-α+IFN-γ synergistically increased levels of endogenous APP and BACE1 in astrocytes, as compared to individual cytokines alone. Following stimulation, astrocytic APP levels reached ~300% of control at 24 h and stayed relatively constant for the duration of the experiment (96 h). BACE1 levels, on the other hand, took longer to increase and gave no indication of leveling off by 96 h when they reached ~400-600% of control. The cytokine combinations also caused significant increases of secreted Aβ40 levels, but this occurred only at 96 h, demonstrating a significant lag period between increased levels of APP and BACE1 on the one hand and elevated Aβ production and secretion on the other. Since levels of both Aβ40 and Aβ42 increase in parallel following BACE1 cleavage of APP [23], it is likely that astrocytic Aβ42 production was also elevated by cytokine combinations including TNF-α+IFN-γ. Unexpectedly, IL-1β treatment resulted in a decrease of secreted Aβ40 levels at 96 h. However, this may be understood in light of the observation that IL-1β treatment did not significantly increase astrocytic APP or BACE1 levels. Along with our results, other reports also indicate that IL-1β may reduce amyloidogenic processing of APP [53,54]. TNF-α+IFN-γ stimulation was associated with robust elevations of APP, BACE1, and Aβ in astrocytes. Interestingly, post-transcriptional mechanisms appeared to be responsible for a large proportion of the TNF-α+IFN-γ stimulated increases in astrocytic APP and BACE1 levels. APP and BACE1 mRNA levels did not increase upon stimulation, with the exception of slightly elevated APP mRNA at 96 h. In fact, BACE1 mRNA levels were significantly decreased by TNF-α+IFN-γ stimulation, strongly suggesting that the BACE1 elevation was post-transcriptional.

Our study is also the first to show that both oligomeric and fibrillar forms of Aβ42 increase the levels of astrocytic APP and BACE1 mRNA and protein, and that they stimulate β-secretase processing of APP in astrocytes. Similar to TNF-α+IFN-γ stimulation, oligomeric and fibrillar Aβ42 treatment of primary astrocytes elevated endogenous APP levels to ~300-500% of control, although these increases were short-lived. Also, Aβ42 oligomers and fibrils caused robust, long-lived increases in astrocytic BACE1 levels (~300% of control), akin to those caused by TNF-α+IFN-γ stimulation. Although we were unable to directly measure Aβ production in Aβ42-stimulated astrocytes, we did interrogate β-secretase processing by analyzing the generation of APPsβsw, the product of BACE1 cleavage, in Aβ42-treated Tg2576 astrocytes. We found that Aβ42 oligomers and fibrils strongly induced astrocytic BACE1 cleavage of APPsw. Given that β-secretase processing of APP and Aβ production are tightly coupled, it is likely that Aβ generation was also elevated in Aβ42-stimulated Tg2576 astrocytes. Finally, the Aβ42-stimulated elevations of astrocytic APP and BACE1 were potentially the result of increased APP and BACE1 gene transcription, at least in part. Although the APP increase was rapid but short-lived, the BACE1 elevation had a slower onset but was sustained for at least 96 h of Aβ42 stimulation.

The TNF-α+IFN-γ- and Aβ42-stimulated increases in astrocytic APP and BACE1 were remarkably similar, but some differences were also observed. For example, the APP and BACE1 elevations appeared to involve both transcriptional and post-transcriptional mechanisms, but to varying degrees depending on the stimulus. The TNF-α+IFN-γ stimulated BACE1 increase was post-transcriptional, since BACE1 mRNA levels were reduced, while the Aβ42-stimulated BACE1 increase involved BACE1 mRNA elevation. In addition, the early phases of the TNF-α+IFN-γ stimulated astrocytic APP elevation did not involve increases in APP mRNA levels, suggesting a post-transcriptional mechanism, while the opposite was true for the Aβ42-stimulated APP increase. Potential post-transcriptional mechanisms could involve enhanced translation or stability of APP and BACE1 mRNAs or proteins, as previously reported in other systems [55-57]. It remains to be determined whether these mechanisms or others could be responsible for the observed elevations of endogenous APP and BACE1 in astrocytes.

**Figure 9:**
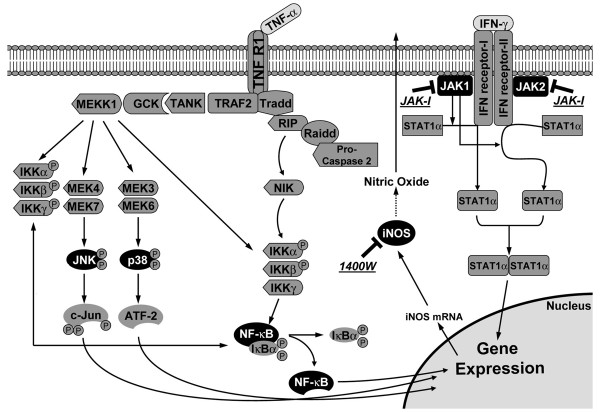
**Schematic diagram of TNF-α and IFN-γ signaling pathways and the inhibitors of signaling molecules investigated in this study**. The common signaling molecules involved in the TNF-α and IFN-γ pathways are indicated. The JAK-I and 1400 W (iNOS) inhibitors are also shown.

To gain insight into the signaling pathways responsible for the TNF-α+IFN-γ-stimulated increases in astrocytic APP, BACE1 and Aβ, we used inhibitors against two signaling molecules known to be involved in neuroinflammation, JAK and iNOS (Figure 9). Except for reducing APP levels with JAK inhibition, blocking neither JAK nor iNOS had a significant effect on astrocytic APP, BACE1, or secreted Aβ40 levels. However, our results do not necessarily mean that these molecules do not play important roles in cytokine-stimulated amyloidogenic APP processing in astrocytes, because the JAK and iNOS signaling cascades have complex regulation and they may adapt to inhibitor treatment [58,59]. Astrocytic effect sizes were largest with cytokine combinations, suggesting that activation of multiple signaling pathways summed together in a synergistic fashion to elevate astrocytic APP, BACE1, and Aβ. Further work using multiple inhibitors or genetic knockdown approaches will be necessary to dissect precisely which signaling molecules are the most critical for cytokine-stimulated elevations of APP, BACE1, and Aβ in astrocytes.

We did not directly address the molecular mechanisms by which Aβ42 raised the levels of APP, BACE1, and β-secretase processing in astrocytes. However, the higher levels of astrocytic APP and BACE1 mRNA that we observed following Aβ42 stimulation suggested increased gene transcription was responsible, at least in part. Little is known about the regulation of APP and BACE1 gene expression in astrocytes. A recent study has suggested that NF-κB may activate the BACE1 gene promoter in TNF-α-stimulated astrocytes [51]. In addition, IFN-γ may activate the BACE1 gene promoter in astrocytes via the JAK/STAT pathway [39]. However, in our study, JAK inhibition did not block the TNF-α+IFN-γ-stimulated increase in astrocytic BACE1, and BACE1 mRNA levels were actually reduced with TNF-α+IFN-γ. The reason of this discrepancy is unknown. Clearly, further work is necessary to resolve this issue in the future.

Far less is known about APP gene regulation in astrocytes. TGFβ appears to increase APP gene transcription in astrocytes, but few other cytokines have been investigated. Regulation of astrocytic APP and BACE1 levels may be complex, since additional evidence exists that pro-inflammatory cytokines may also control the translation of APP and BACE1 mRNA in astrocytes [55,60]. Importantly, except for our work, none of these studies directly addressed whether Aβ42 oligomers or fibrils could increase astrocytic APP or BACE1 mRNA levels.

BACE1 levels in astrocytes are normally very low compared to neurons [23,28]. However, our results have shown that astrocytic BACE1 levels can be strongly induced to ~300-600% over control levels when astrocytes are stimulated by cytokine combinations or Aβ42. Moreover, astrocytic APP levels are also increased several fold by cytokine and Aβ42 stimulation. Together, these effects result in significantly elevated β-secretase processing of APP and Aβ generation in stimulated, as compared to un-stimulated, astrocytes. It has not yet been rigorously determined whether stimulated astrocytes produce similar levels of Aβ as neurons on a per cell basis, but this seems unlikely. However, because astrocytes greatly outnumber neurons, even a relatively small increase in astrocytic Aβ generation may make a significant contribution to the total Aβ burden in the AD brain.

Our study also suggests that a feed-forward mechanism in AD may operate to elevate and sustain astrocytic amyloidogenic APP processing. This feed-forward mechanism may involve the following steps: 1. Pro-inflammatory cytokines including TNF-α and IFN-γ stimulate astrocytes to increase levels of BACE1, APP, and secreted Aβ; 2. As cerebral Aβ levels rise, Aβ42 oligomers and fibrils begin to form; 3. Both oligomeric and fibrillar Aβ42 induce and/or sustain high levels of astrocytic BACE1, APP, and β-secretase processing; 4. Cerebral Aβ levels are further elevated, promoting greater cytokine and Aβ production, thus creating a vicious cycle. Evidence in favor this hypothesis exists, in that Aβ42 is capable of stimulating astrocytes to secrete pro-inflammatory cytokines, and conversely cytokine combinations that include TNF-α and IFN-γ increase astrocytic Aβ synthesis, together forming the elements of a feed-forward loop [35,42-45]. In addition, it is important to note that the BACE1-cleaved ectodomain of APP, APPsβ, is capable of activating microglia [61]. Moreover, Aβ itself can cause microglial activation [3,17]. Thus, microglia are likely to participate in the astrocytic feed-forward mechanism as part of a larger cytokine cycle of neuroinflammation [4,17]. Finally, the trigger of the astrocytic feed-forward loop is unclear, although age-related deficits in Aβ clearance mechanisms may cause an initial rise in cerebral Aβ level that could start the vicious cycle [62,63]. Such an astrocytic feed-forward mechanism could have important implications for both pathogenesis and therapeutic strategies for AD.

## Conclusions

In summary, we demonstrate here that cytokine combinations including TNF-α and IFN-γ, as well as Aβ42 oligomers and fibrils, increase levels of BACE1, APP, and β-secretase processing in cultured primary astrocytes, and that these effects can lead to increased astrocytic Aβ secretion, at least in the case of TNF-α+IFN-γ stimulation. Given that astrocytes are much more numerous than neurons in the brain, our results present strong evidence that activated astrocytes may make a significant contribution to total Aβ burden in AD under neuroinflammatory conditions. Moreover, our data suggest a potential feed-forward vicious cycle of astrocytic activation and Aβ generation. Overall, our results have important pathogenic and therapeutic implications for AD.

## List of abbreviations

**Aβ**: Amyloid-β; **AD: **Alzheimer's disease; **APP: **amyloid precursor protein; **BACE1: **β-site APP-cleaving enzyme 1; **BSA: **bovine serum albumin; **CM: **conditioned media; **CNS: **central nervous system; **DMEM: **Dulbecco's modified Eagle's medium; **DMSO: **Dimethyl sulfoxide; **D-PBS: **Dulbecco's phosphate-buffered saline; **EDTA: **ethylenediamine tetra-acetic acid; **ELISA: **enzyme-linked immunosorbant assay; **FBS: **fetal bovine serum; **GFAP: **glial fibrillary acidic protein; **IFN-γ**: interferon-γ; **IL-1β**: interleukin-1β; **iNOS: **inducible nitric oxide synthases; **JAK: **Janus kinase; **LPS: **lypopolysaccharide; **PVDF: **polyvinylidene difluoride; **RT: **room temperature; **SDS: **sodium dodecyl sulfate; **TNF-α**: tumor necrosis factor-α.

## Competing interests

The authors declare that they have no competing interests.

## Authors' contributions

JZ, TO performed the experiments. JZ, RV conceived of and designed the experimental plan and wrote the manuscript. All authors have read and approved the final version of the manuscript.
